# Effects of the International Temperature Scale of 1990 (ITS-90) on CIE Documentary Standards for Radiometry, Photometry, and Colorimetry^1^

**DOI:** 10.6028/jres.095.042

**Published:** 1990

**Authors:** Klaus D. Mielenz, Jack J. Hsia

**Affiliations:** National Institute of Standards and Technology, Gaithersburg, MD 20899

**Keywords:** CIE Standard Illuminants A, CIE Standard Illuminant D_65_, colorimetry, ITS-90, light sources, photometry, radiometry

## Abstract

The differences between ITS-90 and IPTS-68 above 1235 K are described. It is shown that none of the following CIE definitions or recommendations require revision because of the introduction of the ITS-90: International Lighting Vocabulary definitions; CIE Standard Illuminants A, D_65_, other illuminants; and sources for realizing CIE Illuminants. The effect of the ITS-90 on previously calibrated sources for realizing CIE illuminants is negligibly small.^2^

## 1. Difference Between ITS-90 and IPTS-68

Like its predecessors, the International Temperature Scale of 1990 (ITS-90) [1] is based on numerical values of temperature assigned to a number of defining fixed points and on standard interpolating instruments calibrated at these fixed points. In the radiation temperature region above the freezing point of silver, the ITS-90 can be realized by optical pyrometry and the temperature T_90_ is defined by the equation

$$\frac{L_{\lambda }\left(T_{90}\right)}{L_{\lambda }\left[T_{90}\left(X\right)\right]}=\frac{\exp\left[c_{2,90}/\lambda T_{90}\left(X\right)\right]-1}{\exp\left(c_{2,90}/\lambda T_{90}\right)-1},$$

where *T*_90_(X) refers to any one of the silver [*T*_90_(Ag) = 1234.93 K], the gold [*T*_90_(Au)= 1337.33 K], or the copper [*T*_90_(Cu)= 1357.77 K] freezing points, where *L*_λ_(*T*_90_) and *L*_λ_(*T*_90_(X)) are the spectral concentrations of the radiance of a blackbody at the wavelength (in vacuo) λ at *T*_90_ and at *T*_90_(X), respectively, and where c_2,90_=0.014388 m·K.

In the International Practical Temperature Scale of 1968, Amended Edition of 1975 (IPTS-68) [2] the temperature *T*_68_ was defined by a similar equation:

$$\frac{L_{\lambda }\left(T_{68}\right)}{L_{\lambda }\left[T_{68}\left(\text{Au}\right)\right]}=\frac{\exp\left[c_{2,68}/\lambda T_{68}\left(\text{Au}\right)\right]-1}{\exp\left[c_{2,68}/\lambda T_{68}\right]-1},$$

where *T*_68_(Au)= 1337.58 K and *c*_2,68_=0.014388 m·K.

From the above, the following relationships between the ITS-90 and the IPTS-68 are noted:

1. The value assigned to the second radiation constant, *c*_2_, has not been changed,
  $$c_{2,90}=c_{2,68}=0.014388m\cdot K.$$
2. The ITS-90 gold-point assignment has been lowered by 0.25 K,
  $$T_{90}\left(\text{Au}\right)=T_{68}\left(\text{Au}\right)-0.25K=1337.33K.$$
3. The ITS-90 above the freezing point of silver (1234.93 K) is defined in terms of three fixed points, the freezing points of silver, gold (1337.33 K), and copper (1357.77 K). This introduces a redundancy of the new scale, whereas all previous scales were unambiguously defined in terms of the gold point alone. However, the text of the ITS-90 states that “the *T*_90_ values of the freezing points of silver, gold, and copper are believed to be self consistent to such a degree that the substitution of any one of them in place of one of the other two as the reference temperature *T*_90_(X) will not result in significant differences in the measured values of *T*_90_”.

## 2. Effects on CIE Definitions and Recommendations

The activities of the CIE which relate to temperature and could conceivably be affected by the adoption of the ITS-90 fall in the domain of thermal radiation and its applications in radiometry, photometry, and colorimetry. The published CIE output in these areas can be classified into two broad areas: definitions and recommendations. In what follows, it will be shown that none of these definitions and recommendations requires revision because of the introduction of the ITS-90.

### 2.1 CIE Definitions

#### 2.1.1 General

The output of the CIE contains a large number of definitions of the fundamental concepts, physical quantities, and technical terms used in the areas of light and lighting. These definitions have traditionally been collected in the International Lighting Vocabulary, which is now available in its fourth edition [3] as a joint publication compiled by the CIE in collaboration with the International Electrotechnical Commission (IEC). Insofar as these definitions relate to thermal radiation, they are given in terms of thermodynamic temperatures and are, therefore, independent of the International Temperature Scale and not affected by the adoption of the ITS-90. Specific examples include the definitions of color temperature, correlated color temperature, distribution temperature, radiance temperature, ratio temperature, and similar quantities which are used to assign a single temperature to the spectral power distributions of incandescent or fluorescent lamps, phases of natural daylight, and other sources which are not too different from blackbody sources.

#### 2.1.2 CIE Standard Illuminants

The definitions published by the CIE comprise, as a special class, “standards” in the form of uniquely defined data which form the basis of internationally accepted standard systems. These include “standard illuminants” for applications of colorimetry requiring the use of spectral power distributions which are representative of typical lighting conditions [4,5]. These illuminants are spectral power distributions which are not necessarily realizable by laboratory sources, and are provided by the CIE in the form of numerical tables. At the present time, there are two CIE standard illuminants.

##### Standard Illuminant A

CIE Standard Illuminant A is representative of domestic tungsten lighting and was originally defined as follows [6].

“It is recommended that the following … luminous sources be adopted for the general colorimetry of materials:

“Source A. A gas-filled lamp of color temperature 2848 K …

“For calculations of the spectral energy distribution the constant, *c*_2_, of Planck is taken equal to 14,350 micron degrees.

“Source A. The spectral energy distribution of this source shall be considered for all colorimetric applications as that of a blackbody at the temperature 2848 K.”

This definition was independent of the International Temperature Scale of 1927, which was in effect at the time. After the introduction of the International Temperature Scale of 1948, and once again after the IPTS-68 was introduced, the CIE followed a policy of adopting the assigned values of *c*_2_ but preserving the relative spectral distribution of illuminant A. This was achieved by shifting the color temperature assigned to it, rather than computing new spectral distributions for the original color temperature of 2848 K. Accordingly, the spectral values of CIE standard illuminant A are to be considered independent of the International Temperature Scale and require no adjustments in view of the adoption of the ITS-90. The color temperature assigned to standard illuminant A is a descriptive parameter which depends on the value of *c*_2_ assigned in the International Temperature Scale. Its value is approximately 2856 K on the IPTS-68 and remains the same on the ITS-90 because the value of *c*_2_ has not been changed.

##### Standard Illuminant D_65_

This standard is representative of average daylight and is given in the form of numerical values that have been derived by Judd, MacAdam, and Wyszecki [7] from experimental data taken by others. The tabulated spectral data of illuminant D_65_ are consistent with the IPTS-68 value of *c*_2_, and are also not affected by the introduction of the ITS-90 because *c*_2_ was not changed. Likewise, the correlated color temperature of illuminant D_65_, which is given as 6504 K on the IPTS-68, remains unchanged.

#### 2.1.3 Other CIE Illuminants

The CIE has also defined illuminants which do not have the status of primary CIE standards but are useful for special purposes [5]. Illuminants B (now obsolete) and C represent direct sunlight and average daylight with correlated color temperatures of approximately 4874 and 6800 K, respectively. Three illuminants, D_50_ D_55_. and D_75_, have been defined in addition to standard illuminant D_65_ to represent daylight with approximate correlated color temperatures of 5000, 5500, and 7500 K. Additionally, illuminants F_1_ through F_12_ have been defined to represent typical fluorescent lamps. The published relative spectral distributions of these additional illuminants [5] are consistent, within their estimated accuracies, with IPTS-68 temperatures and, therefore, with ITS-90 temperatures as well.

### 2.2 CIE Recommendations

#### 2.2.1 General

The CIE recommendations [5] concerning the calculation of color temperature and related quantities are dependent on the International Temperature Scale, in that a numerical value of c_2_ is usually specified in these recommendations. However, the change from IPTS-68 to ITS-90 temperatures does not affect any of these recommendations because the value of *c*_2_ remains the same in the new scale.

#### 2.2.2 Recommended Sources for Producing CIE Illuminants

The CIE has recommended specific sources [4,5] which can be used for practical realizations of the spectral distributions defined by CIE illuminants.

For example, the CIE recommends that Standard Illuminant A be realized by means of a gas-filled tungsten-filament lamp known as “Source A” which is operated at a correlated color temperature (2856 K on the IPTS-68) equal to the color temperature associated with Standard Illuminant A. This definition requires no change with the introduction of the ITS-90 because the color temperature associated with Standard Illuminant A has not changed.

At present, no source is recommended for realizing CIE Standard Illuminant D_65_. However, the CIE has recommended a method [8] for assessing sources intended for this purpose. The description of this method does not rely on the International Temperature Scale and, therefore, requires no revision.

Illuminants B and C can be realized by combining Source A with specially formulated liquid filters [5]. The specifications of these filters are independent of the International Temperature scale and require no revision.

There are no CIE recommendations for realizing the D and F illuminants mentioned in section 2.1.3.

## 3. Effects on Calibrated Sources

Although none of the CIE recommendations for laboratory realizations of CIE Standard Illuminants are affected by the adoption of the ITS-90, it should be noted that sources that have previously been calibrated in accordance with these recommendations do not exactly produce the standard spectral distributions. Because the ITS-90 gold-point assignment is closer to the thermodynamic temperature of this fixed point, a source calibrated on the IPTS-68 and operated at a given radiance temperature *T*_68_ will have the ITS-90 kelvin temperature

$$T_{90}=T_{68}-0.25*{\left(T_{68}/1337.33\right)}^{2}$$

[9] and will therefore produce a skewed spectral distribution. For example, a Source-A type lamp having the required correlated color temperature of 2856 K on the IPTS-68 will operate at the ITS-90 temperature 2855 K. Its spectral distribution, normalized to 100.00 at 560 nm, will be 0.07% lower than that of Standard Illuminant A at 460 nm, and 0.05% higher at 660 nm. These differences are small compared to the state-of-the-art calibration uncertainties, so that no adjustments or corrections will be necessary in practice.

## 4. Appendix

### Résumé

Les différences entre ITS-90 et IPTS-68 au delà de 1235 K sont décrites. Il est montré qu’aucune des suivantes définitions ou recommendations de la CIE nécessite de révision à cause de l’introduction du ITS-90: Les définitions du Vocabulaire Internationale de l’Eclairge, les Illuminants Standards A, D_65_, et autres illuminants; et les sources pour la réalisation d’illuminants CIE. L’effet du ITS-90 sur les sources précédement calibrées pur réaliser les Illuminants CIE est néligeablement faible.

### Zusammenfassung

Nach einer Diskussion der Unterschiede zwischen der ITS-90 und der IPTS-68 im Temperaturbereich ueber 1235 K wird gezeigt, dass die Einfuehrung der ITS-90 keine Revision der folgenden CIE Definitionen oder Empfehlungen erforderlich macht: Definitionen des Internationalen Worterbuchs der Lichttechnik; Normlichtarten A, D65, und andere; und CIE Normlichtquellen. Der Einfluss der ITS-90 auf bereits geeichte Normlichtquellen ist vernachlassigbar klein.
